# Fleeting Beauty—The World of Plant Fragrances and Their Application

**DOI:** 10.3390/molecules26092473

**Published:** 2021-04-23

**Authors:** Angelika Kliszcz, Andrzej Danel, Joanna Puła, Beata Barabasz-Krasny, Katarzyna Możdżeń

**Affiliations:** 1 Department of Agroecology and Crop Production, Faculty of Agriculture and Economics, University of Agriculture, Mickiewicza 21 Ave, 31-120 Krakow, Poland; rrpula@cyf-kr.edu.pl; 2 Faculty of Materials Engineering and Physics, Krakow University of Technology, Podchorążych St. 1, 30-084 Krakow, Poland; rrdanela@cyf-kr.edu.pl; 3 Institute of Biology, Pedagogical University of Krakow, Podchorążych 2 St., 30-084 Kraków, Poland; beata.barabasz-krasny@up.krakow.pl (B.B.-K.); katarzyna.mozdzen@up.krakow.pl (K.M.)

**Keywords:** fragrant molecules, essential oils, extraction techniques, food additives, fragrant allelochemicals, biopesticides

## Abstract

This article is devoted to some aspects of the fragrant substances of plant origin applied in the food industry and perfumery as well. Since antiquity many extractive techniques have been developed to obtain essential oils. Some of them are still applied, but new ones, like microwave or ultrasound-assisted extractions, are more and more popular and they save time and cost. Independently of the procedure, the resulting essential oils are the source of many so-called isolates. These can be applied as food additives, medicines, or can be used as starting materials for organic synthesis. Some substances exist in very small amounts in plant material so the extraction is not economically profitable but, after their chemical structures were established and synthetic procedures were developed, in some cases they are prepared on an industrial scale. The substances described below are only a small fraction of the 2000–3000 fragrant molecules used to make our life more enjoyable, either in food or perfumes. Additionally, a few examples of allelopathic fragrant compounds, present in their natural state, will be denoted and some of their biocidal features will be mentioned as an arising “green” knowledge in agriculture.

## 1. Introduction

**Figure 1:**
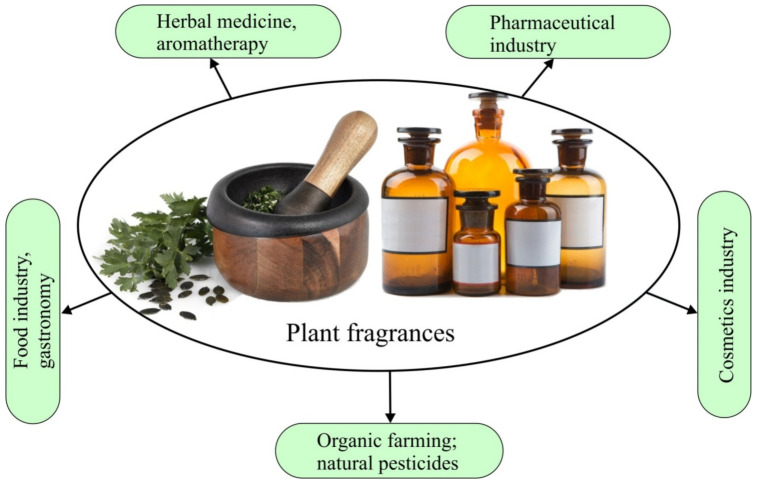
Potential applications of plant fragrances.

It seems that everyone will agree without hesitation that plant-derived chemicals will become an increasing source of inspiration for scientists in the near future. This is due to the large number of plant species that have not yet been fully tested for the content of active substances that can be used as fragrances, food additives, plant protection agents, or new pharmaceuticals. The only question is whether we will make it to that point in light of the systematic destruction of our natural environment. Therefore, it is necessary to make every effort to prevent the extinction of some species as a result of ill-considered human activity. In this article, we would like to focus on the different aspects of using herbal fragrances (Figure 1).

In 1985, a German writer, Patrick Süskind, wrote a novel entitled “*Perfume: The Story of a Murderer*”. The plot of the action focused on an orphan with an unusually developed sense of smell who later became a perfumer capable of the creation of perfume compositions beyond human imagination. In a very short time, this story turned out to be an international bestseller with over 20 million sold copies around the world. In addition to this, 20 years later a film adaptation was premiered. What was the source of success both of the novel and the film adaptation as well? It seems that the reason for this success may be the fact that, from the very beginning, man has been surrounded by smells that provided pleasure and also warned against dangers, such as spoiled food. The aromas surrounding us are extremely diverse so we accumulate countless odour experiences throughout our lives.

**Figure 2:**
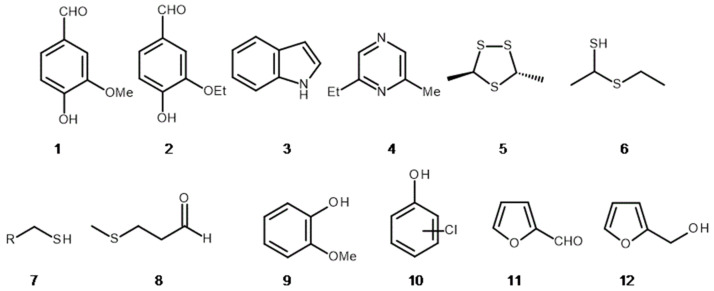
Some food occurring compounds with strong aroma: (**1**) Vanillin, (**2**) ethylvanillin, (**3**) indole, (**4**) 2-ethylpyrazine-6-methylpyrazine, (**5**) (*E*)-3,5-dimethyl-1,2,4-trithiolane, (**6**) 1-(ethylsulphanyl)ethane-1-thiol, (**7**) R=Me, ethanethiol, (**8**) methional, (**9**) guaiacol, (**10**) halophenols, (**11**) furfural and (**12**) 2-hydroxymethylfuran.

Everyone is familiar with the overpowering vanilla scent in ice cream, but it can be found in matured whisky as well. In the first case the vanillin **1** or ethyl vanillin **2** (Figure 2) additives are responsible for this; in the second one, the vanillin is formed due to the decomposition of the lignin structure in oak barrels staves [1]. At the other olfactory pole there are scents we usually avoid, although this is a matter of discussion; *As with poison, the dose makes something poisoning*…, said Paracelsus. A similar situation is observed for fragrances. An indole **3** (Figure 2) in pure form is a white crystalline solid with an offensive odour of faeces [2]. In high dilution it exhibits a pleasant flower smell, so the olfactory experience depends also on the dose. In trace amounts it can be found in orange blossoms or jasmine [3]. Cultural habits are also very important. One of the Chinese traditional foods is stinky tofu. The aroma includes, among others, indole **3** and various sulphur compounds like dimethyl sulphide (CH_3_)_2_S or dimethyl disulphide CH_3_SSCH_3_. The final aroma is perceived as garlic-onion-faecal [4]. Another example can be the fermented Japanese soybean paste—*natto* with the smell of old socks or aged cheese. Even among native Japanese people opinions about this dish are divided, let alone among foreigners. A study by a group of Japanese scientists has shown that pyrazines **4** (e.g., 2-ethylpyrazine-6-ethylpyrazine) and sulphur compounds **5** (e.g., (*E*)-3,5-dimethyl-1,2,4-trithiolane) are responsible for the characteristic smell of *natto* [5] (Figure 2).

The similar situation is observed with mould cheeses (e.g., Camembert) attacking our noses with odour of ammonia NH_3_ and feet, boiled potatoes or sweaty notes represented respectively by isovaleric acid (CH_3_)_2_CHCH_2_COOH, butyric acid C_3_H_7_COOH, methional **8** (Figure 2) [6]. A final example can be the legendary durian fruit (*Durio zibethinus* Murray), although in this case it seems to be more of an urban legend. Some people describe the durian aroma as a mixture of rotten onion, gym socks and garbage stench. The smelliest components are again sulphur compounds, namely, 1-(ethylsulphanyl)ethane-1-thiol **6**, ethanethiol **7** (R=Me) and methanethiol **7** (R=H) (Figure 2) [7]; *De gustibus non disputandum*… so if you do not like it, do not try it. On the other hand, let us enrich our olfactory experience regardless of the smell—especially since the latest research has led to the conclusion that the human nose is able to distinguish unimaginable amounts of fragrance nuances, reaching upwards of one trillion [8]. The period of human life is too short to experience it all, so do not be afraid to experiment with fragrances.

A recent study of Weiss et al. [9] reveals a common olfactory percept, i.e., “white” fragrance, which exists after mixing various equal-intensity components, despite the different scents of each of them individually. Another interesting thing is that odour evaluation is similarly assessed at the individual level but also culturally conditioned. Staszak studied “the geography of scents” and indicated that scents are as important to human beings as the perception of other senses in space [10]. These considerations were also taken up by other fragrance researchers, especially in the context of the city’s odour map [11,12,13].

Another important matter related to the fragrances for human beings is food, i.e., their smell and taste of food. These features can be totally changed by microorganisms or surrounding conditions, e.g., as a result of improper storage (warm and aerobic conditions, as well as moisture) [14]. The developing microbiota enrich food with their metabolites, whose smells and flavours are quite different from the characteristics of the starting material. The species responsible for the formation of unpleasant smells and flavours are, e.g., *Alicyclobacillus acidoterrestris* [15] (spoilage of fruit juices by the production of guaiacol **9** or halophenols **10**—Figure 2), or bacteria from the family Enterobacteriaceae Rahn (smell of rotten meat) [16]. The growth of this bacteria’s family could be limited by the addition of, e.g., 0.02% coriander oil to stored meat [16], while the addition of a bioflavonoid preparation from grapefruit significantly improves sensory features of pork meat during storage, extending its good condition by two days [17].

In the case of the aforementioned thermal treatment, some volatile compounds are lost and the flavour of the resulting product may be different from the starting components. By now, due to the fragrant food additives, we are able to restore these aromas to a certain extent. The addition of some synthetic or natural aromas to food products increases their tastiness and sensory attractiveness. These substances are not always friendly to our health. It happens that during the heat treatment of processed foods a Maillard reaction occurs, resulting in melanoidine molecules (the pigment responsible for the colour of bread crust or roasted meat) and by-products, e.g., furfuryl aldehyde **11**, hydroxymethylfurfural HMF **12** (Figure 2), propene aldehyde H_2_C=CHCHO, acrylamide H_2_C=CHCONH_2_ and others, depending on the pH, substrates and temperature. Most of the low-molecular substances formed at that time have high reactivity, including potential carcinogenicity, such as acrylamide [18]. Nowadays, due to a greater socio-consumer awareness regarding food preservatives, consumers prefer to choose products that are enriched with natural fragrances or processed without the addition of artificial preservatives, e.g., osmotic dehydration [19]. In addition, Piekut [20] proved that besides the olfactory experience, the presence of common spices, such as ginger, dill, lovage and thyme, have very strong antibacterial (on *Escherichia coli* (Migula) Castellani and Chalmers, *Staphylococcus aureus* Rosenbach, *Pseudomonas aeruginosa* (Schröter) Migula, *Bacillus subtilis* (Ehrenberg) Cohn) and antifungal (on *Candida albicans* (C.-P. Robin) Berkhout) properties.

It should be remembered that a substance, to be included in the fragrance group, must be volatile (ability to evaporate at room temperature) and have the ability to interact with olfactory receptors. There are two ways we smell: the orthonasal (directly through the nose) and the retronasal (when the smell gets to the nose from the mouth) routes [21,22]. Chemicals with a mass of less than 300 Daltons bind to proteins on the olfactory receptor neurons (ORNs) at the surface of the olfactory epithelium. Then, the irritation of ORNs produces a gallery of sensory items in the brain that is a representation of the chemical features coming from the external world. Some efforts achieved in gathering all odorants and their retention times received in gas chromatography-olfactometry [23]. In other words, odours, unlike the other sensory inputs, go straight to the limbic system and cerebrum, without processing by the thalamus. That means our bodies respond emotionally and physiologically to scent before we think about it [24]. As a total, 1000 different proteins together encoded by 3% of human genes are located in the olfactory receptors, and these are more responsible for the taste of food than the tastebuds in the mouth, for which Buck and Axel were awarded the Nobel Prize in 2004 [25]. Thanks to these body and brain abilities, nowadays, there are even new methods in medicine for treating people through the sense of smell, called aromatherapy. It is an alternative method of healing and strengthening the body, using the olfactory channel to stimulate the brain in a different way than medications or physical therapies can [26].

It happens that, in some cases (e.g., benzaldehyde **17**), the same compound can be found as a food additive, a perfume ingredient or allelopathic substance—three functions within one structure. Therefore, this short review is devoted to plant-derived aromatic substances applied in food and the perfume industry as well. Additionally, a few examples of allelopathic fragrant compounds, present in nature, will be denoted and some of their biocidal features will be mentioned.

## 2. Preparation Techniques of Essential Oils

For centuries, plants have been an endless source of aromas as evidenced also by the Bible’s Song of songs e.g., “Spikenard and saffron; calamus and cinnamon, with all trees of frankincense; myrrh and aloes, with all the chief spices… Be awake, O north wind; and come, O south, blowing on my garden, so that its spices may come out”. For this reason, man tried to isolate the essence of their fragrance: the essential oils (EOS). These substances belong to the naturally fragrant materials widely applied in perfumery, pharmacy, food industry and as agrochemicals as well. The oldest method of obtaining EOS, olive oil or citrus oil is cold pressing. This method dates to ancient times and it is relatively cheap. Both raw materials are available in large quantities. For example, in 2008 the annual production of orange and lemon oils were estimated on the level of 51,000 and 9200 metric tonnes, respectively [27].

**Figure 3:**
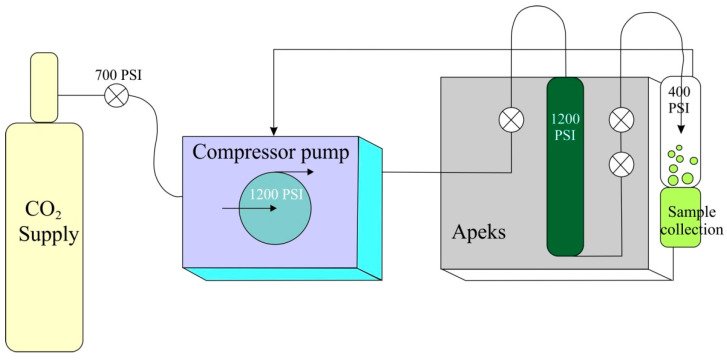
Supercritical fluid extraction (SFE) of essential oils (according Yousefi et al., 2019 [28]; modified).

Another equally old technique is *enfleurage*. It is an extraction of volatile substances from plants with cold animal fat like lard or tallow. After leaving for a few days, plant material is removed and replaced with the fresh one. This procedure is repeated several times. Finally, the fat saturated with essential oils is mixed with alcohol in which aromatic substances are dissolved and the fat is separated off. After evaporation of the alcohol solution an absolute is obtained. This technique was applied to flowers very sensitive to temperature, like jasmine, tuberose or orange. It was used in ancient Egypt and in France. The variation of this procedure is an application of hot fat (60–70 °C). Due to the high costs cold and hot *enfleurage* was abandoned and replaced with more effective solvent extraction with ethyl alcohol, low-boiling hydrocarbons, aliphatic esters or florasol (1,1,1,2-terafluoroethane). This procedure is applied for the preparation of jasmine absolute. The disadvantage of this extraction can be the residues of extractive solvents in the final product. This can be avoided by using extraction with a supercritical fluid (SFE) with a solvent like butane, propane, ethylene or carbon dioxide. On the industrial scale it is used for extraction of caffeine from coffee and tea and hop essential oils [28] (Figure 3).

Additionally, the SFE technique is used in the extraction of sunflower, jathropa and sesame oil [29]. Supercritical carbon dioxide was also applied in isolation of EOS from many plant materials, like *Mentha spicata* L., *Coriandrum sativum* L., *Ocimum basilicum* L. or *Eucalyptus globulus* Labill. [28,30,31,32,33].

**Figure 4:**
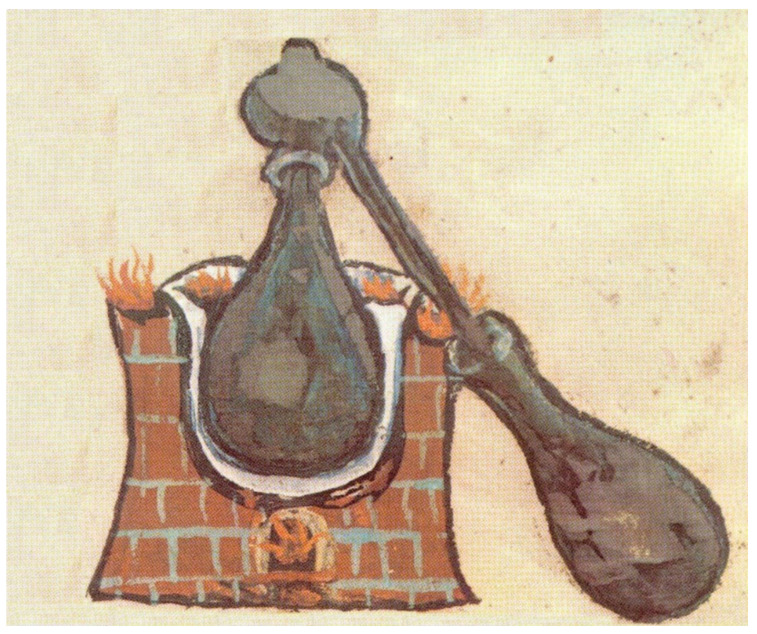
An ancient distillation apparatus—alembic (lat. *alembicus*); scheme of an alembic from a medieval manuscript [35] (modified).

Finally, some *distillation techniques* should be mentioned. The distillation process was invented in the Middle East ca. 1200 BC and over time adapted for perfumery purposes [34] (Figure 4).

The ingredients of EOS are sensitive to temperature and the direct distillation may cause their decomposition and it can’t be applied. The solution is the *steam distillation*. In the classic version plant material is placed in an alembic over boiling water on the perforated tray. The passing steam removes volatile substances. The distillate is collected in a receiver and, due to the insolubility of EOS in water, the upper (or lower layer) of EOS can be easily separated. The collected material can be subjected to fractional distillation and rectification under reduced pressure. In *hydrodistillation* method plant materials and water are heated together in the alembic and vaporised essential oils and steam are condensed like in the previous method. This process can be supported with microwave heating of the plant material/water mixture for more effective isolation of aromatic substances [36,37,38].

The disadvantages of the abovementioned method are the prolonged time of extraction, hydrolytic decomposition of extracted compounds (e.g., esters) and solvent residues in the final material. Lucchesi and co-workers [39] developed a new technique named *solvent-free microwave extraction* (SFME), in which the fresh plant material was irradiated in a microwave oven without any solvent. Dry seeds were moistened prior to use. The volatiles were collected in a Clevenger apparatus. The authors reported significant time and energy reduction in comparison with the classical hydro-distillation procedures. Microwave-assisted chemical reactions settled permanently in the chemistry laboratory, but there is a constant need for new techniques in the laboratory and in industry as well. Such an example is the application of ultrasound in laboratory practice in organic and analytical chemistry [40,41]. Some authors have used it in extractions of antioxidants, dyes or essential oils from plant material [42,43]. Just recently a review devoted to this technique was published [44]. EOS, after blending or diluting with solvents (water or alcohol), are given common names following their respective concentrations: *Eau de perfumes* (10–20% of essential oils), *eau de cologne* (3–5% of perfume in alcohol/water) and *eau de toilette* (less than 2% of perfume) [45].

## 3. The Hidden Ingredients of EOS

**Figure 5:**
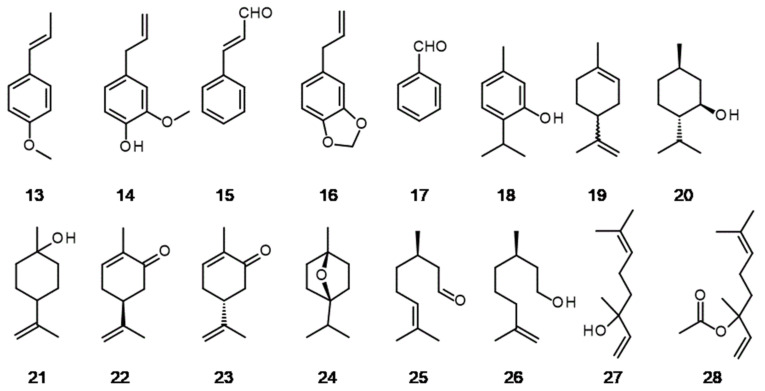
Some of natural isolates applied as food additives or perfume ingredients: (**13**) *trans*-anethole, (**14**) eugenol, (**15**) cinnamaldehyde, (**16**) safrole, (**17**) benzaldehyde, (**18**) thymol, (**19**) limonene, (**20**) menthol, (**21**) terpineol, (**22**) *R*-carvone, (**23**) *S*-carvone, (**24**) eucalyptol, (**25**) citronellal, (**26**) rhodinol, (**27**) linalool, (**28**) linalyl acetate.

Olfactory experiences of floral scents are mirrored by the chemical diversity of volatile organic compounds (VOCs) emitted by plants around the world. EOS in food as fragrances or antioxidants, part of the fragrant composition in perfumes or agrochemicals, are complicated mixtures of many VOCs. The significant majority of them are terpene hydrocarbons and terpenoides (their monofunctional derivatives). Moreover, it can be found in numerous aromatic and aliphatic esters, aromatic aldehydes, phenols, ketones, ethers and heterocycles. There are too many examples of the great wealth of ingredients in essential oils to list them here. In some cases, the content of aromatic substances in essential oils is so high that it is profitable to separate them, either by distillation or fractionated freezing. These isolates are widely applied in food, pharmaceutical and chemistry industry. Some representative examples of isolates are depicted in Figure 5 [46,47,48]. Gourmets from all over the world know the Chinese five spice powder, which is a mixture of cloves (*Syzgium aromaticum* (L.) Merr. and Perry), fennel (*Foeniculum vulgare* Mill.), Sichuan pepper (*Zanthoxylum bungeanum* Maxim.), Chinese cinnamon (*Cinnmomum cassia* (L.) J. Presl) and star anise (*Illicium verum* Hook. f.) for preparing dishes based on duck, chicken and pork. Among the many aromas, the slightly sweet smell of the latter is clearly visible. The compound responsible for this aroma is *trans*-anethol **13** (Figure 5).

In pure form it can be extracted from anise (*Pimpinella anisum* L.), anise star or fennel essential oils either by steam distillation or by extraction with supercritical carbon dioxide [49,50]. The annual production of anise essential oil reaches several hundred tons and, due to the high content of anethole, there is no need for its chemical synthesis. For example, the predominant components of essential oil from *P. anisum* are *trans*-anethole 95% and methyl chavicol 2% [51,52]. Anethole is used as flavouring additive in the food industry for candies, chewing gums and baked goods. In many cases, the addition of anethole emphasizes the so-called brown aromas, such as chocolate, coffee, tea and root beer [53]. Moreover, it is applied in many alcoholic beverages like German *Jägermaister* or Spanish *anisado*. A significant part of the world’s production of anethole is used in cosmetic products such as soaps, toothpastes and mouthwashes [54].

Visitors to dental offices know the characteristic clove scent that accompanies them during dental procedures in the mouth. A substance belonging to the phenol group eugenol **14** is responsible for this intense aroma (Figure 5). Eugenol exhibits antiseptic properties. It can be obtained from cinnamon leaf oil or clove oil. The content of eugenol in clove oil reaches 81% and, taking into account the fact that the production of clove oil reaches 100,000 tons per year, chemical synthesis is not profitable [52,55]. Clove oil is used in blackberry, cherry and smoke flavour composition in the food industry [52]. Moreover eugenol is used in perfumery for the creation of clove, carnation, oriental and spicy notes. In addition to its fragrance properties, eugenol has been tested for its antimicrobial, anti-inflammatory, anti-cancer and anti-stress properties. Detailed information on this can be found in a review article published by Khalil et al. [45]. Another source of intense aroma in the five spice powder is cinnamon. The best known species are Ceylon cinnamon (*Cinnamomum verum* J.Presl) and cassia (*C. casia*). The compound responsible for the smell in both cases is cinnamaldehyde **15** (Figure 5). Cassia essential oil typically contains 85% cinnamaldehyde and is used for flavouring cola drinks and a component of cherry, vanilla and nut flavours [52]. Production of *E*-cinnamaldehyde is mainly based on steam distillation of the oil from the bark of the genus *Cinnamomum* e.g., *C. zeylanicum* Blume, where the content of this aldehyde reaches 97%. The leaves of the same tree are a rich source of eugenol [32]. Cinnamaldehyde has been used as a food additive for over 100 years because of its tantalizing smell. It is used to flavour bread, cakes, puddings and drinks. Thus far, it is considered a safe food additive. Some studies indicate that this compound is nephrotoxic, at least in rats [56]. Another representative in our gallery of aromatic food additives is safrole **16**, a compound with a Janus face (Figure 5). This is an example of a sudden end to long-term use of a compound that was considered safe. It occurs, inter alia, in the North American plant *Sassafras albidum* (Nutt.) Nees, from which the essential oil is obtained by steam distillation. Until 1960, safrole was used to flavour root beer, various soft drinks, chewing gums and toothpaste. It owed its popularity to the aroma that was referred to as the fragrance of a ‘candy store’. After 1960, it was found to be weakly hepatocarcinogenic and banned as a food additive [57]. To make matters worse, safrole is a precursor to the synthesis of psychedelic substances, e.g., MDMA. On the other hand, safrole is applied in the synthesis of valuable compounds used in perfumery and for pesticide formulations.

The simplest aromatic aldehyde, benzaldehyde **17**, is probably one of the most frequently used natural flavouring substance and is second only to vanillin **1**, which is also an aldehyde, but in most cases its synthetic equivalent is used instead of a natural compound (Figure 5). They are influenced by economic considerations. Benzaldehyde can be obtained from a variety of natural sources, including fruit stones or by chemical synthesis. The content of benzaldehyde in apricot fruit stone is ca. 1.2%. This material is a main source of natural benzaldehyde [52]. Due to the intense aroma of almonds, it is used to flavour cakes, fruit juices, soft drinks, hard and soft candies and chewing gums. In some cases this compound is applied as a flavour enhancer in beers. It is also included in many food fragrances, e.g., cherry and almond [58]. Benzaldehyde found an application as a repellent for driving bees to get them away from a honeycomb [59].

Thymol **18** is a crystalline substance with an intense odour of thyme and belongs to the group of compounds called phenols. In *Thymus vulgaris* L. and *T. zygis* L. essential oil the content of this phenol varies between 22% and 50% [60] (Figure 5). Thyme oil is used as a flavouring agent in food products, such as sweets, syrups or as a major use in the form of seasoning blends [52]. It should be mentioned that, in addition to the inherent aromatic properties, thyme oil is also characterised by strong antioxidant properties, which is also used in the food industry [60].

Limonene **19** is the most important and most abundant monoterpene in nature. It is the main ingredient of many essential oils obtained from citrus fruits. Its content in some cases reaches almost 90–95%, hence, they are its main source. Given the enormous amounts of citrus fruits processed into juice each year, limonene production is measured in tens of thousands of tons. It is obtained by steam distillation of the residue from this process. It has a pleasant lemon scent. In the food industry, limonene contained in essential oils is used as a flavouring in the production of desserts, ice cream and non-alcoholic beverages. One of the new trends is adding pure terpenes to cocktails—limonene is among them [61].

Natural menthol **20** is isolated from EOS either of *Mentha ×piperita* L. or *M. arvensis* L. by freezing technique (Figure 5). The content of menthol in EOS from *M. ×piperita* reaches 75% so it’s the most significant source of natural compound. The biggest producers are China and India [62]. Menthol was a popular additive to cigarettes, but it seems that it will share the fate of safrole and will be banned in the tobacco industry, not least because it does not encourage young people to experiment with cigarettes [63].

Again we can quote a Latin saying “de gustibus non disputandum”: There is no disputing about taste. This saying seems to fit very well with another natural aroma found in food, terpineol **21** (Figure 5). It is one of the main flavours of *lapsang suochong* tea, in addition to longifolene terpene. Responsible for its occurrence is the drying process, where pine smoke is used. Not all consumers are satisfied with the slightly smoked aroma of this tea, reminiscent of smoked bacon or some peated whiskey from Scotland [64]. Only small amounts of natural terpineol are extracted from essential oils as an isolate. The vast majority is produced from natural pinene by chemical synthesis or biotechnological methods [65]. Terpineol is widely used in fragrant composition (e.g., lime), household products (e.g., air fresheners, washing powders). This compound is also an insect attractant [66].

The two enantiomeric varieties of carvone are an elegant example of the structural influence on the olfactory properties of a compound. *R*-Carvone **22** has a mint scent while its mirror image, *S*-carvone **23**, smells like caraway (Figure 5). Both enantiomers can be obtained from natural sources like caraway seeds or spearmint oil, respectively. Both spices have been known since ancient times as references to them are already found, for example, in the Bible in the Book of Isaiah. *R*-Carvone is used in chewing gum industry (e.g., Wrigley spearmint) to enhance the impact and extend the duration of the fruit flavour [14]. Both carvones are applied in alcohol beverages industry, too. *S*-carvone is responsible for aroma in German *Kümmel* liquor or Scandinavian *akvavit*. Spearmint containing *R*-carvone is an ingredient of many mixed drinks and mint flavoured liquors, e.g., in Spain in *The Old Liquor Store Spearmint Liqueur*.

Eucalyptol (or cineol) **24** is a colourless liquid with a camphor odour, found mainly in the essential oil from eucalyptus (Figure 5). One of the most abundant sources of cineol are leaves of *Eucalyptus globulus*. Its content is over 60% accompanied with pinene up to 22% [67]. Essential oil is used as flavouring additive in various food products like baked goods, hard candies, meat products and beverages. Due to fresh odour this substance is applied in large quantities in oral hygiene products.

Citronellal **25** is a main constituent of Citronella essential oil from Java or Ceylon (Figure 5). The oils are produced via steam distillation of fresh or dry herb (*Cymbopogon nardus* (L.) Rendle or *C. winterianus* Jowitt ex Bor). Both oils differ in terms of smell. Java oil has a strong lemon scent, while Ceylon oil has a citronellal one. Due to their very strong aroma, both oils are used in low doses in products such as alcoholic beverages, breakfast cereals, jelly, soft drinks and various candies [68]. Citronellal is a starting material for the synthesis of hydroxy citronellal (cosmetic fragrance) and insect repellent *p-*menthane-3,8-diol [69]. Rhodinol **26** is colourless liquid with a pleasant, rose-like scent. It can be obtained from geranium or citronella (*C. nardus*) essential oils by fractional distillation (Figure 5). Rhodinol is approved as a flavouring ingredient and is used for the composition of floral and rose notes.

Linalool **27** is an unsaturated aliphatic alcohol belonging to the terpenes group (Figure 5). It exists in two enantiomeric forms. *S*-Linalool has a pleasant floral scent. In turn, the fragrance of *R*-linalool is dominated by wood and lavender accents. It can be obtained as an isolate from essential oils of *Lavandula angustifolia* L. and *L. latifolia* Medik. In recent years there has been a growing interest in the application of essential oils to traditionally-used preservatives. Just recently Hussein et al. [70] investigated the effect of linalool and piperine on the quality of chicken meat stored in refrigerated conditions. Both compounds retarded the growth of Gram-positive and Gram-negative bacteria and ensured the stabilization of meat colour parameters. Linalyl acetate **28** is a colourless liquid with a floral, fruity scent (Figure 5). It can be obtained from lavender oil (*L. angustifolia*), whose main ingredients are linalool (37.11%) and linalyl acetate (34.96%) [71]. Together with linalool these compounds are substances applied for flavouring Earl Grey teas [52]. Both compounds can be also isolated from bergamot essential oil. Bergamot oil is used in the production of candies. The most famous are traditional Turkish *Akide* hard candies considered to be Turkey’s culinary heritage and amber-coloured French *Bergamotes de Nancy*. The last candies are produced from sugar and glucose syrup with bergamote oil. However, the largest amounts of linalyl acetate are consumed by the perfume industry, where it is used for many fragrance compositions, such as lavender, ylang-ylang and fancy. Linalyl acetate is more volatile than linalool and is used for the creation of a fresh top-note to a fragrance.

**Figure 6:**
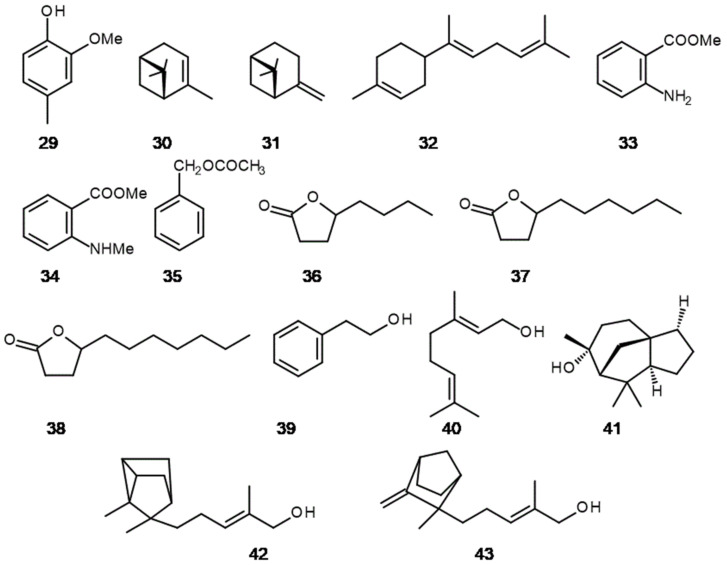
Natural isolates applied as food additives or perfume ingredients: (**29**) 4-methylguaiacol, (**30**) α-pinene, (**31**) β-pinene, (**32**) α-bisabolene, (**33**) methyl anthranilate, (**34**) methyl *N*-methylanthranilate, (**35**) benzyl acetate, (**36**) γ-octalactone, (**37**) γ-decalactone, (**38**) γ-undecalactone, (**39**) 2-phenylethanol, (**40**) geraniol, (**41**) cedrol, (**42**) α-santanol, (**43**) β-santanol.

Earlier mentioned guaiacol **9** is a valuable raw material for the production of two aromatic substances, vanillin and eugenol. Natural guaiacole can be extracted from forest creosote obtained mainly from beech. Guaicol content in a beech tar distilled at 200–210 °C reaches 26.5% [72]. It is accompanied by 4-methylguaiacole, also known as creosol or valspice **29**, with a content of 35% (Figure 6). Both derivatives can be found in whiskey aroma, especially from Islay Island. The characteristic smoky smell comes from the malt dried with burning peat. People who do not drink alcohol may experience a similar aroma experience when drinking *Lapsang suochong* tea. Its leaves are dried over burning pine wood. The tea has an aroma that is similar to the previously mentioned types of whiskey.

Several plants contain α- and β-pinene (**30**, **31**), which are related to herbal, turpentine or pine forest odour [73] (Figure 6). Considerable amounts of α-pinene compound can be found in *Helianthus tuberosus* L. through GC-MS analysis, i.e., 25% of all substances in hydrodistilate (unpublished results). This perennial plant from the Asteraceae Dum family until now was studied only as a valued plant in the functional food and alcohol industry (tubers with a high content of inulin) and cultivated for energetic purposes (vast amounts of annual aboveground biomass with a satisfying calorific value of 15.93 MJ∙kg^–1^) [74]. Pure α-pinene is easily obtained from turpentine oil. It is a starting material for further synthesis of borneol and camphor (67). Another fragrant compound can also be extracted from *H. tuberosus*, and there is β-bisabolene **32**, which have balsamic odour, and that plant contains significant amounts of these constituents in leaves, 40% *w*/*w* (unpublished results) (Figure 6). Yeo et al. [75] stated that β-bisabolene exhibited selective cytotoxic activity for mouse cells and human breast cancer cells, and Kusuhara et al. [76] proved that α-pinene is responsible for inhibiting tumour growth in mice. Moreover, α-pinene and β-bisabolene are both included as a flavouring substances specified Annex I to Regulation (EC) No 1334/2008 of the European Parliament and of the Council regarding food additives (in force) [77].

Methyl anthranilate **33** is applied in the food industry to aromatise candies, soft drinks, chewing gums and alcoholic drinks (Figure 6). It exhibits fruity grape aroma. It is isolated from essential oil of Petitgrain mandarin (*Citrus reticulata* Blanco), where its content reaches 50%. Due to the high demand of this flavouring agent and to avoid the synthesis from petrochemical components, some efforts to produce it from natural components were undertaken. Luo et al. [78] applied microbial procedures starting from glucose and *Escherischa coli* and *Corynebacterium glutamicum* strains.

The anthranilic acid derivative is another ester, namely methyl *N*-methyl anthranilate **34**, which is abundant in *Citrus reticulata* (Figure 6). This plant, and specifically the essential oil from the leaves, is also the main source of this raw material, which is used in the perfume industry for shampoos, toiletries and household cleaners. It is also a raw material for the synthesis of the previous anthranilic ester by the microbiological *N*-demethylation of the *N*-methyl group [79].

Another ester that is a natural isolate is benzyl acetate **35** (Figure 6). Although the ester is very easy to obtain from benzyl alcohol and acetic acid, the natural product is far superior to its synthetic counterpart in terms of fragrance depth. It is obtained by fractionated distillation of *ylang-ylang* oil derived from flowers of *Cananga odorata* (Lam.) Hook.f. and Thomson. Its content varies between 5.5–17.5%. To quote an opinion on benzyl acetate from Hermitageoils (Europe’s Specialist Supplier of Naturals): “This incredibly beautiful material is the result of a fractional distillation of ylang ylang flowers and it shows perfect harmony with jasmine,” [80] nothing more, nothing less. Benzyl acetate is widely used in the perfume industry. The scent of peach fruit is another example of the sources of new fragrances, including γ-decalactone **37**, for a variety of uses (Figure 6). When looking for the possibility of purchasing this compound for perfumery or food purposes, one can find offers where sellers claim that this compound is a natural isolate from peach fruit. It seems, however, that it is currently a niche production and the price of such a product is correspondingly high. In the 1980s, it was over USD 10,000 per kilogram. Due to the significant demand for this compound, reaching several hundred tons per year, its entire industrial production is based on biotechnological methods based on ricinoleic acid, which allowed to reduce the price of this lactone to USD 300 per kilogram [81,82].

γ-Decalactone is used as food additive in peach, apricots and strawberry flavours. Another important natural lactones are γ-octalactone **36** and γ-undecalactone **38** (Figure 6). Both of them exhibit coconut flavour and are used for coconut, oakwood, cream and peach notes. Both natural isolates are extracted from coconut palm [83].

2-Phenylethanol **39** can be isolated in pure form via fractional distillation of rose essential oil or through various synthetic routes (Figure 6). It has a pleasant rose-like floral odour. Natural alcohol as an isolate is used in food industry as a flavouring agent, but for perfumery application it is synthesized or produced by microbial fermentation from L-phenylalanine [81]. In rose essential oils 2-phenylethanol is accompanied by terpene alcohol geraniol **40** (Figure 6). Moreover, this compound can be found in other EOS like geranium, palmarosa, *Cymbopogon martini* (Roxb.) W.Watson or *Monarda fistulosa* L. var. *menthifolia.* In the last case, the content of this compound in EOS is over 95% [84]. The demand for this compound is relatively high and is estimated over 1000 tons per year. It is widely applied both in the perfumery industry and in domestic and household products [85]. Due to the high demand for geraniol, it is also obtained by semi-synthetic methods from other natural isolates, such as α-pinene, β-pinene, linalool or citral. It is impossible to list all isolates that are obtained from plant material. Finally, cedrol **41**, α-santanol **42** and β-santanol **43** can be mentioned (Figure 6). Cedrol is obtained from Texas cedarwood oil and is a precursor for other fragrant substances for the cosmetic industry, like cedryl acetate [67]. It seems that for some time the raw material for the production of this compound will be available. The situation is somewhat different with sandalwood oil and santalols—its main components. Due to the overexploitation of sandalwood, its resources are diminishing, especially in India and Indonesia. Sandalwood oil can be obtained from trees that are at least 30 years old. In order to meet the growing demand for sandalwood oil, sandalwood plantations have been established, for example in Australia. The current price of the oil is nearly USD 100,000 per ton. Sandalwood EOS is widely applied in perfumery as a fixative [67].

Some fragrances are used for medicinal purposes as well. They act not only as a biocidal agents [16,86,87], but also as agents carrying healing effect in alternative ways for medicaments, i.e., the olfactory path. Aromatherapy, the developing branch of modern medicine [76,88,89,90,91,92,93], started in ancient times. Chinese medicine used, e.g., citrus, eucalyptus and sandalwood oils as main healing fragrant factors. Egyptians treated with myrrh, thyme, lavender, rose, peppermint, aloe and almond oils. Romans and Greeks prepared fragrant substances mainly from rose, lavender and peppermint. For example, the major element of essential oil of *Eucalyptus* ssp. (*E. globulus* and *E. citriodora* Hook.) 1,8-cineole **24** is used for stimulation of respiration to relieve anxiety [94]. Treating persons through the sense of smell is an alternative way of healing and strengthening the body, using the olfactory channel to stimulate the brain in a different way than medications or physical therapies can.

## 4. The View from the Nature Side—Allelopathy

The other side of the coin of the fragrant substances is their allelopathic nature. Every chemical substance in the natural environment has more or fewer allelopathic properties, which, according to the definition of allelopathy, are substances produced by all living components of biocenosis (donors) interacting with each other (acceptors) with a multitude of chemical links. The active fragrant substances can be distributed primarily by plants [95], but also by microorganisms (like acetoin which is commonly associated with the smell of yogurt and produced through PGPR-Plant Growth Promoting Rhizobacteria [96], or others [97,98]. Liu and Zhang [99] provide an interesting review on the effects of bacterial volatile emissions.

From living aboveground plant parts two main processes could occur: volatilization and leaching processes [100]. The first one is a source of volatiles (essential oils), mainly terpenes, which are released from specialised morphological structures, and the second one occurs when raindrops reach the leaf and wash away a larger amount of substances soluble in water, like organic acids, phenols, glycosides, alkaloids or flavonoids. Additionally, underground parts of plants release exudates, which very easily penetrate into the soil solution thanks to its hydrophilic character (through osmosis) or persist near the roots as a hydrophobic molecules [100,101,102]. Moreover, injured plant parts also serve as a source of fragrant allelochemicals, like pericarp of citrus, or leaves of lemon balm (*Melissa officinalis* L.), peppermint, or aloe (*Aloe vera* (L.) Burm. f.), whose juice was used by women in ancient Egypt to perfume their bodies [103]. 

Sometimes to obtain a fragrant substance there is the necessity to decay the plant (trees from genus *Aquilaria* (agarwood) and *Gyrinops*, Thymelaeaceae family) through mold (like Ascomycetous mold, *Phaeoacremonium parasitica* (Ajello, Georg and C.J.K. Wang) W. Gams, P.W. Crous and M.J. Wingf.) [104]. This process occurs naturally (e.g., mechanical injury of tree, as well as a result of insect activity) or it is initiated by man (by buried wood pieces in the moist ground to decay) [105]. The oil or resin appears afterward and its scent is known for centuries in Jewish, Indian, Chinese or Persian societies, and still highly valued now. Additionally, *Aquilaria* trees are endangered and protected, and agarwood scent cannot be synthesized, although chemical substitutes already exist, but do not come even close in mimicking the natural product [104]. One tola (11,664 g) of agarwood oil costs in Q1 2020 on average USD 450 [106]. Examples of some other fragrant allelochemicals are exhaustively presented in paper Bali et al. [107].

The highest quantity of fragrant substances is located in the following parts of plants: leaves and seeds (or fruits), and in flowers, as well. As mentioned above, the morphology of these organs is created in precisely the way as to contain secretory cells and spaces, that store secreted substances. Secreting cells produce large amounts of special metabolism products, thus they characterize features associated with intense metabolism: Dense cytoplasm, well-developed endoplasmic reticulum and Golgi apparatus, large cell nuclei with a high content of DNA, occurring often as a polyploid type. Secretory units can accumulate bubbles with fragrant substances internally (finally mainly in vacuoles) or externally. The second way is most frequently used by plants. It occurs when secretory cells are located in some areas of epiderma and secreting fragrant substances through the cell wall. This process is realized by terpenoid glandular trichomes located on the skin surface [108], like in, e.g., hop plants [109], peppermint [110] or through accumulating secreting substances between secretory cells (like in citrus fruits epiderma, where these spaces are formed by the breakdown of secretory cells, or pine resins, which are accumulated in developing regular channel galleries between secretory cells). Interesting is that not only has genetics influenced the chemical composition of aroma compounds in plants but, in basil leaves (*Ocimum basilicum*), it may change after various colours of artificial mulch have been applied during cultivation. Loughrin and Kasperbauer [111] found that the change of the ratios of red, blue and far-red light reflected to growing plants can influence both leaf morphology and chemistry. These leaves developing over red surfaces had greater area, moisture percentage, and fresh weight than those developing over black surfaces. Interestingly, basil plants grown over yellow and green surfaces produced significantly higher concentrations of aroma compounds than those grown over white and blue covers.

**Figure 7:**
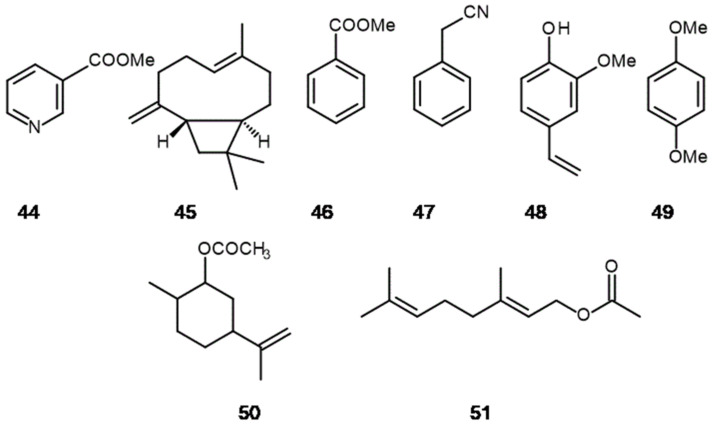
Structures of some allelopathic compounds: (**44**) methyl nicotinate, (**45**) β-cariophyllene, (**46**) methyl benzoate, (**47**) phenylacetonitrile, (**48**) 2-methoxy-4-vinylphenol, (**49**) 1,4-dimethoxybenzene, (**50**) dihydrocarveol acetate, (**51**) geranyl acetate.

Another common method to let the fragrant substances appear is through nectaries activity, where the secretory functions are performed by the epidermis (or trichomes) or cells below it. Nectar is also secreted by external walls into the intercellular spaces, from which it is extracted outside through special stomata. They are located in generative (floral) as well as vegetative parts of plants (extrafloral) [112]. Fragrant substances secreted through the nectaries are crucial for reproduction of all entomophile plants and serve as communicator agents with mutualist pollinators, or antagonists, such as florivores and herbivores. Examples of following fragrant compounds can be found in nectaries: (*S)*-(+)-linalool **27**, methyl nicotinate **44**, β-caryophyllene **45**, methyl benzoate **46**, or phenylacetonitrile **47** [113,114,115] (Figure 7). Other specialized structures providing fragrant substances in plants are osmophores present in the following families: mainly Araceae Juss. and Orchidaceae Juss., but also Iridaceae Juss., Passifloraceae Juss. ex Kunth in Humb., Fabaceae Lindl., Solanaceae Juss, Apocynaceae Juss. [116,117]. According to fragrant volatiles from nectars, Burdon et al. [118] found an interesting relation between plants and bacteria colonizing floral tissue of *Penstemon digitalis* Nutt. ex Sims. (Plantaginaceae Juss.). Since bacterial activity negatively affects the quality and quantity of nectar of this plant, they employ chemical (allelochemical) agents to suppress the bacteria colonies in nectar tissue. (*S*)-(+)-linalool **27**, which is emitted from nectar (but not from petals), reduces bacteria density. Moreover, this compound only affects the bacteria isolated from nectar (bacteria isolated from leaves were not affected). On the other hand, Junker et al. [115] found that some bacterial strains (Enterobacteriaceae), originating from flowers *Saponaria officinalis* L. (Caryophyllaceae Juss.), are adapted to native volatiles more than those settled on leaves, although isolates from petals characterize with less microbial diversity (both at the family and genus levels).

Among the common role of scent, namely positively affects pollinators and seed fitness [119], there are reverse trend in Nature, too. Sometimes fragrant scent (e.g., 2-methoxy-4-vinylphenol **48**) attracts not only pollinators, but also florivores (like in orchid *Dichaea pendula* (Aubl.) Cogn.) [120] (Figure 7). Compound **48** is also responsible for “clove” odour of some beers like *Weisbier* and *Wit* [121]. The study of Theis and Adler [122] confirms that florivory as well as pollination has shaped the evolution of floral scent, and indicate the pronounced role of compound 1,4-dimethoxybenzene **49** in this mechanism (Figure 7).

Summarizing, many factors influence floral scent emission. In addition to the environmental features, like temperature [123], moisture [124], soil water content [125], or light intensity [126], there are a few biotic factors affecting this process, like plant pollination strategies [127], bacterial or fungal occurrence [118], insect invasion [128], density of plants or the presence of other plant species [129]. Moreover, the ecology of plants indicated that they secreted fragrant substances not only to attract pollinators, but also for defence against pests (florivore, herbivore and microbial agents). Therefore, the fragrant molecules play a dual role in plant ecology.

## 5. The Fragrant Danger—Examples of Biopesticides

Plants have had 400 million years to develop highly complex chemical defences against specific predators, and extracts from plants have been used by humans for at least 2000 years to deter or kill plant pests. It so happens that substances that are toxic to plant pests usually also have a specific smell. Extracts [130,131], decoctions and macerates are used for this purpose, but essential oils, as the most concentrated form of allelochemicals isolated from plants, are a promising alternative to chemical substances used in agriculture or for food processing. In 2009, the EU Commission issued Directive 2009/128/EC [132] on the sustainable use of pesticides, which also promotes the use of alternative, environmentally friendly strategies in pest management. However, besides the very long and expensive legal procedure, the main problem of commercialization of such substances of natural origin is the need to standardize the procedure that gives a unified product’s content, which is not always easy taking into account the need to diversify the supply of raw material, its genetic variability [133] and its specific responses to changing environmental conditions during the growing season [119]. The difficulty of applying them into fields (thanks to its volatility and non-solubility in water) can be overcome, however, by microencapsulating technology and using adjuvants [134].

In a time of accelerating “green” knowledge in agriculture, more and more scientific papers are devoted to biocidal opportunities for EOS against plant pests. According to plant-plant relations, EOS are studied mainly for their inhibitory effects on seed germination (weed management) [135,136]. However, studies on their foliar application around the world, as well as anti-weed mulch activity, are also performed. Synowiec [137] correlated highly phytotoxity-essential oils (from *Carum carvi* L., *Mentha ×piperita*, *Thymus vulgaris* plants) with their chemical content, which consists of compounds from the group of oxygen derivatives of monoterpenes (64–95% of content), in particular monoterpenic alcohols, esters and ketones. The laboratory tests showed promising activities of these EOS against weeds, mainly the small-seed ones, i.e., *Amaranthus retroflexus* L. and *Centaurea cyanus* L. The common maize weed, *Echinochloa crus-galli* (L.) P. Beauv. is also susceptible to caraway oil (being an oil-in-water emulsion at a dose of 2.5%) after foliar application [138]. Laosinwattana et al. [139] found that *Tagetes erecta* L. essential oil completely inhibited seed germination *of that weed* too, due to inhibiting α-amylase activity (at 2 mL·L^−1^ emulsifiable concentrate of this essential oil), and, with concentrations of 10–80 mL·L^−1^ post-emergence of foliar application, caused wilted and desiccated leaves of this weed. Interesting examples of Australian flora endemics pretending to be novel essential oil-yielding crops with their anti-pest capabilities are cited by Sadgrove and Jones [140].

Another example is provided by Martins et al. [141], which found that direct application of eucalyptus essential oils (at concentrations between 2.5–20.0 µL·mL^–1^ and between 1.25–5.00 µL·mL^–1^) in a terrestrial system may provoke immediate and short-term deleterious effects on soil organisms, e.g., Collembola Lubbock and common leaf colonizers, i.e., *Alternaria alternata* (Fr.) Keissl., *Fusarium roseum* Link, *Mucor hiemalis* Wehmer, *Penicillium* sp. and *Penicillium glabrum* (Wehmer) Westling, respectively, which affects the capability of soil agroecosystem to decompose organic matter. The main substances of Eucalyptus oil scent are: β-Citronelal (71.77%, *Eucalyptus citriodora*), Dihydrocarveol acetate **50** (50.82%, *E. gamphacephala* DC.), Geranyl acetate **51** (50.49%, *E. macarthurii* H.Deane and Maiden) and (+)-Limonene (28.82%, *E. staigeriana* F.Muell. ex F.M.Bailey) [142] (Figure 7).

Another way to use aromatic plants biomass in agriculture is mulching fields, both with living mulch and processed biomass derived from fragrant plants. The latter means planting aromatic plants as stubble or cover crops after a cash crop and leaving whole biomass in the soil, planting with another plant or for the winter season. The fragrant residues primarily inhibit the weed occurrence. This practice is, for now, used in agriculture of tropical climates due to the common occurrence of aromatic species in natural conditions. Dhima et al. [143] study an anti-weed effect of mulch from seven annual aromatic plants and determined that the number of barnyard grass plants emerging from anise, lacy phacelia and common balm green manure plots was lower by 47%, 49% and 50%, respectively (compared with green manure-free plots). Moreover, in oregano and lacy phacelia-green manure-herbicide-untreated plots, maize silage yield was 33–34% greater than that in the green manure-free herbicide-untreated plots. In the research of landscape mulches aromatic plants (mainly trees and shrubs) play a pivotal role. However, a few features of them are monitored to meet the needs of urban architecture (i.e., retain or changing their colour, rate of decomposition). Among the studied species, Duryea et al. [144] defined the longest-lasting colour (i.e., not changing during a year) as ‘reddish brown’ offered by pine bark mulch. Other mulches (from *Eucalyptus*, *Melaleuca*, Pine needles) transformed their colour more or less in shades of grey during the year. Mulch from Cypress transforming its colour every three months (starts from pink, then reddish yellow, pink, light brown, and again pink after one year).

In addition to the living mulch approach, there is also another one, focused on the use of fragrant plants’ biomass derived from various processes (like distillation, etc.) and it assumes incorporating into the soil the aromatic plants’ residues from the EOS-obtaining industry or pharmaceutical procedures. A comprehensive review on this topic was provided by Saha and Basak [145]. Farmers can benefit from aromatic and medicinal plant cultivation in terms of diversification of farm incomes, which is in line, on the other hand, with the path of rediscovery of ancient traditions to cultivate these plants in traditional regions like the Piedmont Region (Italy). Cultivation of rosemary, thyme and lavender has been spreading there in marginal areas [146].

## 6. Conclusions

The plant kingdom, serving humankind for centuries, represents a vast reservoir of compounds with fragrant properties and proven biological traits. How can the fragrant substances save the world? Since, by observing widespread use of toxic (long-term persist and overused) chemical solutions in agriculture [147,148], an evident negative impact on the many biocenosis all over the world (their homeostasis, maintaining and biodiversity), as well as the development of herbicide-resistant weeds, and human and animal health, the promotion of sustainable practices of local communities based on natural active compounds are highly appreciated and now observed. Examples of essential oils with biocidal features presented above can become the driving force of the supply side in the agricultural industry, especially improving agricultural technology for the organic branch.

This very short review gives a small glimpse into the application of fragrant substances of plant origin across a variety of industries. At present, a total of 2000–3000 flavouring chemicals, both natural and synthetic, are used commercially, and various fragrant substances are used as pest management control agents. The search and analysis of natural fragrant substances are especially challenging due to a future of sustaining agriculture practices and production of healthy food, as well as biocenotic homeostasis. Some of them have very promising medicinal features in treating depression, pain, and microbiologically-based diseases. On the other hand, the rapid progress in modern analytical techniques gives birth to the hope that many new aromatic molecules will be isolated and characterised in the future. Moreover, we have to remember that a large number of organic chemists from academic and industrial laboratories constantly modify known structures and synthesise new molecules with interesting fragrant properties, which do not exist in nature, so we can expect new olfactory experiences.

Reaching the origin of fragrant substances it should be mentioned that the role of the enormous number of plant-released chemicals are important in mediating the interaction between plants and their environment, across every space of the whole living world. Since consumers and farmers are orienting towards eco-friendly products, the reduction of chemical inputs is pursued. In the era of coronavirus, it remains only to express the belief that the sense of taste and smell will never be taken away from humanity.

## Data Availability

Not applicable.

## Abbreviations

EOS	Essential Oils
EU	European Union
EC	European Commission
GC-MS	Gas Chromatography—Mass Spectrometry
HMF	Hydroxymethylofurfural
MDMA	Methylenedioxy-methylamphetamine (CAS-42542-10-09)
ORNs	Olfactory receptor neurons
SFE	Supercritical fluid extraction
SFME	Solvent-free microwave extraction
VOCs	Volatile organic compounds
